# *In Silico* Knockout Studies of Xenophagic Capturing of *Salmonella*

**DOI:** 10.1371/journal.pcbi.1005200

**Published:** 2016-12-01

**Authors:** Jennifer Scheidel, Leonie Amstein, Jörg Ackermann, Ivan Dikic, Ina Koch

**Affiliations:** 1 Molecular Bioinformatics, Institute of Computer Science, Johann Wolfgang Goethe-University Frankfurt am Main, Frankfurt am Main, Germany; 2 Institute of Biochemistry II, Johann Wolfgang Goethe-University Hospital Frankfurt am Main, Frankfurt am Main, Germany; 3 Buchmann Institute for Molecular Life Sciences, Frankfurt am Main, Germany; Centre for Research and Technology-Hellas, GREECE

## Abstract

The degradation of cytosol-invading pathogens by autophagy, a process known as xenophagy, is an important mechanism of the innate immune system. Inside the host, *Salmonella* Typhimurium invades epithelial cells and resides within a specialized intracellular compartment, the *Salmonella*-containing vacuole. A fraction of these bacteria does not persist inside the vacuole and enters the host cytosol. *Salmonella* Typhimurium that invades the host cytosol becomes a target of the autophagy machinery for degradation. The xenophagy pathway has recently been discovered, and the exact molecular processes are not entirely characterized. Complete kinetic data for each molecular process is not available, so far. We developed a mathematical model of the xenophagy pathway to investigate this key defense mechanism. In this paper, we present a Petri net model of *Salmonella* xenophagy in epithelial cells. The model is based on functional information derived from literature data. It comprises the molecular mechanism of galectin-8-dependent and ubiquitin-dependent autophagy, including regulatory processes, like nutrient-dependent regulation of autophagy and TBK1-dependent activation of the autophagy receptor, OPTN. To model the activation of TBK1, we proposed a new mechanism of TBK1 activation, suggesting a spatial and temporal regulation of this process. Using standard Petri net analysis techniques, we found basic functional modules, which describe different pathways of the autophagic capture of *Salmonella* and reflect the basic dynamics of the system. To verify the model, we performed *in silico* knockout experiments. We introduced a new concept of knockout analysis to systematically compute and visualize the results, using an *in silico* knockout matrix. The results of the *in silico* knockout analyses were consistent with published experimental results and provide a basis for future investigations of the *Salmonella* xenophagy pathway.

## Author Summary

*Salmonellae* are Gram-negative bacteria, which cause the majority of foodborne diseases worldwide. Serovars of *Salmonella* cause a broad range of diseases, ranging from diarrhea to typhoid fever in a variety of hosts. In the year 2010, *Salmonella* Typhi caused 7.6 million foodborne diseases and 52 000 deaths, and *Salmonella enterica* was responsible for 78.7 million diseases and 59 000 deaths. After invasion of *Salmonella* into host epithelial cells, a small fraction of *Salmonella* escapes from a specialized intracellular compartment and replicates inside the host cytosol. Xenophagy is a host defense mechanism to protect the host cell from cytosolic pathogens. Understanding how *Salmonella* is recognized and targeted for xenophagy is an important subject of current research. To the best of our knowledge, no mathematical model has been presented so far, describing the process of *Salmonella* Typhimurium xenophagy. Here, we present a manually curated and mathematically verified theoretical model of *Salmonella* Typhimurium xenophagy in epithelial cells, which is consistent with the current state of knowledge. Our model reproduces literature data and postulates new hypotheses for future investigations.

## Introduction

Inside the host, *Salmonella enterica* serovar Typhimurium, in the following referred to as *Salmonella*, can invade phagocytic and non-phagocytic cells, such as intestinal epithelial cells. After invasion of epithelial cells, the bacterium resides and starts replication inside the *Salmonella*-containing vacuole (SCV). A fraction of the bacteria does not stay inside the SCV. The SCV membrane becomes damaged, and *Salmonella* gets access to the host cytosol and starts to replicate with high rates [1–3]. To protect the host cell, *Salmonella* rapidly becomes a target of xenophagy.

Xenophagy, also known as antibacterial autophagy, is a process of capturing and eliminating cytosolic pathogens, like *Salmonella*. Xenophagy is an early defense mechanism against pathogens that enter the cytosol [4]. Cytosolic bacteria are targeted with ubiquitin or galectin-8 [4–6], leading to the recruitment of the autophagy receptors, p62 [7], nuclear dot protein 52 kDa (NDP52) [8], and optineurin (OPTN) [9]. Autophagy receptors simultaneously bind ubiquitin- or galectin-8-labeled *Salmonella* as well as the autophagic protein of the microtubule-associated protein 1 light chain 3 (LC3)/*γ*-aminobutyric acid receptor-associated protein (GABARAP) family to recruit LC3/GABARAP-positive autophagosomal membranes [10]. Then, the bacteria are engulfed by an isolated membrane termed phagophore. The phagophore elongates and forms a double-membrane compartment, the autophagosome. The autophagosome can fuse with lysosomes, resulting in the destruction of the bacteria in the autolysosome. Defects in this defense pathway of xenophagy are linked to chronic inflammation, like Crohn‘s disease [11–16].

The xenophagy pathway has recently been discovered, and the exact molecular details are still not completely understood. Many bacteria have been shown to be targeted for the xenophagy pathway. Until now, *Salmonella* is the best-studied model organism for xenophagy. Neither quantitative nor qualitative mathematical models, describing the process of *Salmonella* xenophagy, have been presented so far. Existing literature provides a rich repertoire of molecular interaction information. To compile the known information and to analyze the biological system of xenophagy, we developed a mathematical model of *Salmonella* xenophagy in HeLa cells.

We applied the Petri net (PN) formalism [17] to analyze the structure of the model and the basic dynamics of the system, even without explicit knowledge of the kinetic data. PN have been successfully employed to study signaling pathways and regulatory networks [18–23]; for an overview see [17, 24]. The model recapitulates in detail the molecular mechanisms of *Salmonella* xenophagy, including the galectin-8 and ubiquitin-dependent targeting of cytosolic *Salmonella* and of *Salmonella* inside the damaged SCV. Additionally, the model comprises regulations like nutrient-dependent regulation of autophagy and TANK-binding kinase 1 (TBK1)-dependent activation of the autophagy receptor, OPTN. The mechanism of TBK1 activation in HeLa cells upon *Salmonella* infection is not known, but it has been shown that a knockdown of TBK1 increases *Salmonella* replication in HeLa cells [8, 9, 25]. To model the activation of TBK1, we proposed a new mechanism of TBK1 activation, suggesting an autoactivation by TBK1 oligomerization.

We analyzed invariant properties to check the model for consistency and biologically meaningful behavior [17]. To observe the model behavior and the corresponding biological effect, we performed *in silico* knockouts of specific proteins, e.g., NDP52, OPTN, and TBK1. We compared the model behavior with published knockout and knockdown experiments to verify the biological credibility of the model. Knockouts that has not been experimentally investigated yet provide new hypotheses for further studies. This can help to plan further experiments more precisely.

## Methods

Mathematical modeling is an iterative process, see S1 Fig. The iterative process consists of four steps: literature research, model development/modification, model analysis, and model verification. Initially, we performed an extensive literature research to manually collect functional information about *Salmonella* xenophagy. Due to the fact that the xenophagy pathway was recently discovered, no data are currently available in public databases, such as the Kyoto Encyclopedia of Genes and Genomes (KEGG) [26]. We started with a small Petri net model based on a few references. Then, we checked the model structure for consistency, correctness, and completeness on the basis of PN theory [17]. The mathematical analysis based on PN theory was essential to detect model structures that contradict the biological knowledge. To resolve the identified inconsistencies, we repeatedly studied the literature and modified the wrongly extended model structure. If no further inconsistencies were found, step by step, we extended the Petri net by functional information from the literature based on experiments, including, among others, yeast two-hybrid assays, pull-down assays, co-immunoprecipitation, knockdowns, knockouts, and X-ray crystallography. S2 Table gives a complete list of the literature references we used to build the network model of the xenophagy pathway. The iterative process of network validation, correction, and extension terminated with a PN model, which contains all known processes of *Salmonella* xenophagy in epithelial cells at the current state of research.

### Petri nets

Due to the well-established analysis techniques, the expandability to a quantitative model, and the intuitive graphical representation of PN, the PN formalism is widely used for modeling and simulating biological systems. In the following, we give a short explanation of PN terms and methods that are necessary to understand the study. For a more comprehensive introduction to the formalism and analysis techniques of PN, we refer to Koch et al. 2011 and Reisig 2012 [17, 27].

PN are directed, bipartite, labeled graphs, consisting of two types of nodes, places and transitions, connected by directed, weighted arcs. Places, modeling the passive part, represent biological substances, such as proteins or protein complexes. Transitions, representing the active part, stand for chemical reactions, such as binding reactions, dissociations, or degradations. A transition is called *input transition* if the transition has only outgoing and no incoming arcs. A transition is called *output transition* if the transition has only incoming and no outgoing arcs. Places and transitions are graphically represented as circles and rectangles, respectively. Arcs are labeled by integer numbers, which correspond to the stoichiometric factors of the biochemical reactions.

Places can carry movable objects called *tokens*, which can move along the arcs by firing of transitions and reflect the system’s dynamics. The distribution of tokens over all places is called *marking*. The marking of the model defines the *state* of the system. The system’s state before any transition has fired is called *initial state* with an *initial marking*. A token can have different meanings. Tokens may represent a discrete amount of chemical substances, e.g., one molecule or number of organisms as *Salmonella*. A token on a place may alternatively represent a fulfilled precondition of a reaction. Such a precondition could be, e.g., the availability of a substance above a given threshold level.

To simplify the graphical layout of the PN, we applied *logical places* for substances participating in many reactions. Logical places are identical graphical copies of a biological substance involved in various reactions of the model.

To design, analyze, and simulate the PN, we used the open-source tool MonaLisa [28]. We structurally verified the model by the analysis of invariant properties called *transition invariants* (T-invariants) and *place invariants* (P-invariants) [17]. Mathematically, a T-invariant represents a set of transitions, whose firing reproduces an arbitrary state of the system. Biologically, a T-invariant describes a functional module at steady-state. Each T-invariant has to represent a biological meaningful subpathway of the system. We verified the biological interpretation of each T-invariant. If each transition is a member of at least one T-invariant, the PN is *covered by T-invariants*.

Mathematically, a P-invariant represents a set of places, for which the weighted number of tokens remains constant. Each P-invariant corresponds to the conservation of a biological substance, e.g., of an enzyme that is neither synthesized nor degraded in the network. We checked the biological interpretation of each P-invariant. For a more strict verification, we deleted all input and output transitions of the PN and checked the biological interpretation of the P-invariants of the resulting subnetwork. The subnetwork without input and output transitions has much more P-invariants than the original and should be *covered by P-invariants*, i.e., each place is a member of at least one P-invariant. A biological PN should be connected, covered by T-invariants, and each of the invariants has to have a biological meaning [20, 24].

### *In silico* knockout analysis

Once we had constructed and verified a model, we performed *in silico* knockout analyses to examine the model behavior for perturbations of specific proteins. If a transition is knocked out, all T-invariants that contain the corresponding transition are affected. We performed single as well as double knockout experiments. We visualized the results of the knockout experiments using a *sensitivity matrix*. The rows and columns indicate the proteins of the model, whose synthesis, i.e., input transitions, has been knocked out. The entries represent the influence of the knockout experiments on the system. This influence is measured by the percentage of the affected T-invariants—a measure for the model’s sensitivity to perturbations. The diagonal of the matrix shows the influence of the single knockouts on the system. To investigate the influence of a knockout on proteins or protein complexes, we introduce a new concept of *in silico* knockout analyses based on T-Invariants and visualize the results in the *in silico knockout matrix*. The rows of this matrix represent a certain knockout, e.g., the deletion of an input transition. The columns represent the proteins or protein complexes, which may be affected by the knockout. The entries of the matrix are binary values encoded by either a red or a green circle. An entry is green, if the substance can be produced under steady-state conditions, i.e., the substance is the outgoing place of a transition that is part of a T-invariant. An entry is red, if the substance can not be produced under steady-state conditions, i.e., the substance is not the outgoing place of a transition that is part of a still functional T-invariant. Biologically, a green entry indicates that the knockout has no effect on, e.g., the production of a substance or a protein complex, and a red entry represents a negative effect, e.g., the substance or the protein complex can not be produced anymore.

**Fig 1:**
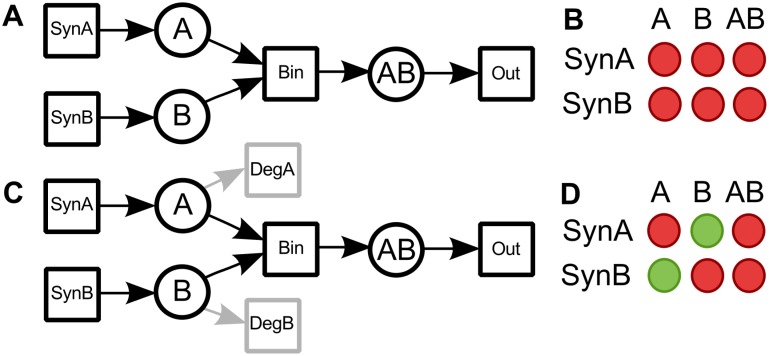
*In silico* knockout matrix and the corresponding PN. (A) A small PN, consisting of three places, protein *A*, protein *B*, and a protein complex *AB*, and four transitions, *SynA*, *SynB*, *Bin*, and *Out*, which describe the synthesis of *A*, the synthesis of *B*, the binding of both proteins, and the outflow of the complex to the environment, respectively. (B) The *in silico* knockout matrix of the PN shown in part A. The matrix has a row for each input transition, *SynA* and *SynB*, and columns for each substance, *A*, *B*, and *AB*. The binary values of an entry are color-coded by either a red or a green circle. An entry becomes red, if the corresponding place is not the outgoing place of a transition that is part of a still functional T-invariant. The entry is green, if the corresponding place is still the output place of a transition that is part of a T-invariant. For the PN in part A, the knockout of either *SynA* or *SynB* is sufficient to have a negative effect on all substances, *A*, *B*, and *AB*. Consequently, all entries in the matrix are red. (C) The PN is modified by adding the output transitions, *DegA* and *DegB*. Now, the knockout analysis becomes more specific. The knockout of *SynA* blocks the production of *A* and complex *AB*, but *B* is not affected. Vice versa, the knockout of *SynB* leaves *A* unaffected. (D) The corresponding *in silico* knockout matrix of the modified PN shown in part C.

Fig 1 exemplifies the concept of *in silico* knockout matrices. The example PN in Fig 1A describes the synthesis of two proteins, *A* and *B*, the binding of these proteins to form a complex, *AB*, and the outflow of the complex to the environment. The T-invariant consists of the reactions *SynA*, *SynB*, *Bin*, and *Out*. In this example, the syntheses of *A* (input transition *SynA*) and *B* (input transition *SynB*) are knocked out. The resulting *in silico* knockout matrix (see Fig 1B) visualizes the influence of these knockouts on *A*, *B*, and *AB*. The rows of the matrix represent the input transitions, *SynA* and *SynB*, and the columns the substances, *A*, *B*, and *AB*.

The knockout of either the syntheses of *A* (input transition *SynA*) or *B* (input transition *SynB*) is sufficient to have a negative effect on all substances in the model, *A*, *B*, and *AB*. Consequently, all entries in the matrix are red. However, we would expect that the synthesis of protein *B* has no effect on protein *A* and vice versa. We correct this effect by the addition of output transitions for *A* and *B* (see Fig 1C). Biologically, these output transitions, *DegA* and *DegB*, represent the degradation of the proteins. The additional output transitions generate two new T-invariants, which separately include the synthesis and the degradation of *A* and *B*, respectively. The first T-invariant consists of the transitions *SynA* and *DegA*, and the second T-invariant of *SynB* and *DegB*. Now, *B* is not affected by a knockout of *A* and vice versa, see Fig 1D. The concept of sensitivity and *in silico* knockout matrices is directly related to *Mauritius maps*, which compute and visualize the impact of knockouts as a binary tree structure [19].

## Results and Discussion

### Biological model of *Salmonella* xenophagy

**Fig 2:**
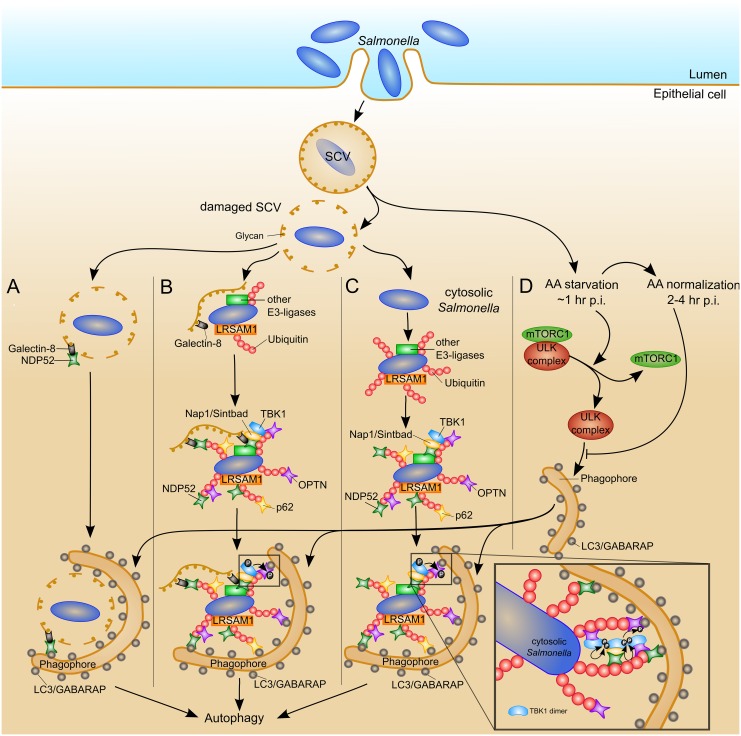
Schematic model of *Salmonella* xenophagy. Inside lumen of the mammalian small intestine, *Salmonella* invades epithelial cells and resides within the SCV. A fraction of the bacteria does not persist inside the SCV and enters the host cytosol. Before *Salmonella* gets cytosolic, *Salmonella* is inside a damaged SCV and is partially exposed to the cytosol. (A) Galectin-8 can bind to host glycans, which are normally hidden inside the SCV. Galectin-8 can target damaged SCV to the autophagic pathway by binding the autophagy receptor, NDP52. (B) Besides galectin-8, ubiquitin is an important eat-me signal for targeting *Salmonella* inside a damaged SCV to the autophagy pathway. *Salmonella* gets ubiquitinated by the E3 ubiquitin ligase, LRSAM1, and other E3 ubiquitin ligases. The autophagy receptors, p62, NDP52, and OPTN, serve as adaptors, which can bind both ubiquitin-chains and LC3/GABARAP molecules on phagophores. The binding affinity of OPTN with LC3 can be enhanced by TBK1 phosphorylation of OPTN. The hypothetical mechanism for TLR4-independent activation of TBK1 is depicted in a detailed view. TBK1 is recruited via the Nap1/Sintbad-NDP52 complex or the Nap1/Sintbad-NDP52 complex and OPTN to ubiquitinated *Salmonella*. This recruitment induces a high local concentration of TBK1 dimers, resulting in their oligomerization and autophosphorylation. (C) *Salmonella* that is already fully cytosolic, is only targeted by ubiquitin to the xenophagy pathway and not by galectin-8. (D) Autophagy is induced by intracellular amino acid (AA) starvation at 1–2 hr post infection (p.i.), which is assumed to be triggered by the damage of the SCV. The intracellular AA starvation results in the inhibition of mTOR—a subunit of mTORC1. Under AA starvation conditions, mTORC1 is inactivated and dissociates from the ULK1 complex, recovering the kinase activity of the ULK1 complex. The activated ULK1 complex seems to be required for the phagophore formation. The intracellular AA pool normalizes 3–4 hr p.i., and mTORC1 localizes at the surface of the SCV and gets reactivated.

We proposed a model of *Salmonella* xenophagy illustrated in Fig 2. To get a comprehensive overview of the current state of research, we included all known processes that target *Salmonella* to xenophagy in epithelial cells.

Inside the lumen of the mammalian small intestine, *Salmonella* infiltrates epithelial cells, see Fig 2 upper part. Once inside the cell, most of the bacteria stay in the SCV. Inside the SCV, *Salmonella* has non-ideal life conditions, such as an acidic environment [29] with limited space and likely nutrient-poor [30]. A small fraction of *Salmonella* disrupts their vacuolar membrane of the SCV [4, 31], and enters the cytosol. Inside the cytosol, *Salmonella* has ideal life conditions, such as access to many nutrients, sufficient space, and a neutral pH. Under these conditions, *Salmonella* replicates inside the cytosol at a higher rate than inside the vacuole [1–3].

#### Galectin-8-dependent *Salmonella* xenophagy

Before *Salmonella* escapes into the cytosol, *Salmonella* is inside a damaged SCV and is partially exposed to the cytosol. It is assumed that both *Salmonella* inside a damaged SCV and cytosolic *Salmonella* are targets of xenophagy [4, 32]. The damage of the vacuolar membrane exposes host glycans to the cytosol, which are normally hidden inside the SCV. The cytosolic host protein, galectin-8 [6], can bind to these beta-galactoside-containing glycans, see Fig 2A. Galectin-8 can target damaged SCV to the autophagic pathway by binding of NDP52 [6, 33].

#### Ubiquitin-dependent *Salmonella* xenophagy

Besides galectin-8, ubiquitin is another important eat-me signal. *Salmonella* with cytosolic access gets immediately surrounded by a dense coat of polyubiquitin chains [5]. Ubiquitin seems to target *Salmonella* inside a damaged SCV, see Fig 2B, as well as cytosolic *Salmonella*, see Fig 2C. The E3 ubiquitin ligase leucine-rich repeat and sterile *α* motif-containing-1 (LRSAM1) can catalyze the ubiquitination of cytosolic *Salmonella* [34, 35]. Whether proteins of the *Salmonella* surface are ubiquitinated, ubiquitinated host proteins attach and accumulate to the *Salmonella* surface, or E3 ubiquitin ligase itself gets autoubiquitinated, is still debated in ongoing research. LRSAM1-ubiquitination favors K6 and K27 linkages [34]. It is known that *Salmonella* is surrounded by a ubiquitin coat, containing multiple linkage types, at least K48, K63, and linear ubiquitin chains [36, 37], suggesting that in addition to LRSAM1, other unknown E3 ubiquitin ligases are involved in the ubiquitination of *Salmonella*, which are yet to be discovered.

After labeling *Salmonella* with ubiquitin, adaptor proteins bind to the ubiquitin chains to carry the bacteria to the autophagic pathway termed autophagy receptors. NDP52 [8], p62 [7], and OPTN [9] are known autophagy receptors for *Salmonella* xenophagy. They can bind both ubiquitin around the *Salmonella* surface and LC3/GABARAP. All three autophagy receptors are non-redundant and are recruited independently of each other to the same bacteria. The receptors are necessary to protect the cell from cytosolic, high-replicating *Salmonella* [7–9, 38]. The depletion of two of the three autophagy receptors leads to a non-additive effect on the restriction of *Salmonella* replication [9, 38]. This behavior indicates that all three autophagy receptors participate in the same pathway and have distinct functions, in addition to the binding of ubiquitin and LC3/GABARAP.

Beside its role as autophagy receptor, NDP52 recruits TBK1 to ubiquitinated bacteria in a complex with the adaptor proteins, Nap1 or Sintbad [8]. Also, the function of OPTN is characteristic. The LC3 binding affinity can be enhanced by OPTN phosphorylation via TBK1 [9, 39, 40]. Activated TBK1 phosphorylates OPTN at serine-177 [9]. OPTN can bind LC3/GABARAP through its N-terminal LIR motif, adjacent to serine-177. Phosphorylated OPTN at serine-177 enhances the binding affinity with LC3B by changing the hydrogen bond formation [9]. The increased binding affinity promotes xenophagy of cytosolic *Salmonella*.

#### TBK1-dependent enhancement of *Salmonella* xenophagy

TBK1 is a serine/threonine-protein kinase, which can be activated via Toll like receptor 4 (TLR4) signaling. The pattern recognition receptor, TLR4, is a component of the innate immune system and can be activated in response to *Salmonella* lipopolysaccharide (LPS). HeLa cells do not express a functional TLR4 due to the missing of the accessory protein, MD2. Nevertheless, HeLa cells treated with TBK1 siRNA showed an increased *Salmonella* replication [8, 9, 25]. In HeLa cells, TLR4 signaling seems not to be required to restrict bacterial proliferation, indicating an unknown way to activate TBK1.

To model the activation of TBK1 in epithelial cells, we proposed a new hypothetical mechanism for TLR4-independent activation of TBK1, see Fig 2. This mechanism suggests a spatial and temporal regulation of this process. We assume that the TBK1 activation is a multistep mechanism. Inactive TBK1 exists as a dimer, which can be activated by autophosphorylation in trans through high-order oligomerization of TBK1 dimers [41–44]. In the inactive state, without oligomerization, autophosphorylation is prevented, because the two kinase domains are located on the opposite sides of the dimer [43]. TBK1 is recruited via the Nap1/Sintbad-NDP52 complex to ubiquitinated *Salmonella* [8]. Additionally, TBK1 can bind to OPTN, which is also recruited to *Salmonella* via ubiquitin chains. We assume that the recruitment of TBK1 by NDP52 or by both autophagy receptors, NDP52 and OPTN, plays a role in bringing the TBK1 dimers close together. Additionally, K63-linked ubiquitination of TBK1 may provide spatial proximity. This high local concentration of TBK1 dimers leads to TBK1 oligomerization into high-order complexes, which allows its autophosphorylation in trans. In support of this hypothetical mechanism for TLR4-independent activation of TBK1, NDP52 and OPTN are localized in the same area around *Salmonella*, separately from p62 [9, 38]. It would be interesting to examine, whether TBK1 shares these subdomains around *Salmonella* together with NDP52 and OPTN. A further evidence is that optimal TBK1 activation seems to require the interaction between OPTN and K63-polyubiquitin or linear-polyubiquitin [45]. Additionally, Nap1 facilitates the oligomerization of TBK1 in 293T cells [46]. Further studies are required to validate the proposed hypothetical mechanism for TLR4-independent activation of TBK1, which stimulates xenophagy in HeLa cells.

#### Nutrient-dependent regulation of *Salmonella* xenophagy

It was recently shown that the induction of *Salmonella* xenophagy seems to be regulated by the intracellular amino acid (AA) level [47, 48], see Fig 2D. AA starvation is triggered by bacteria that get access to the cytosol, resulting in xenophagy induction. The AA starvation is not a result of the consumption of the bacteria for their metabolism. It is assumed that the damage of the SCV membrane triggers a host-driven AA starvation at approximately 1–2 hr p.i. The intracellular AA starvation results in the inhibition of the protein kinase mammalian target of rapamycin (mTOR). mTOR forms a complex with Raptor and mLST8/G*β*L, the mTOR complex 1 (mTORC1). At normal AA level, active mTORC1 is associated with the UNC-51-like kinase 1 (ULK1) complex, leading to the inhibition of the ULK1 complex kinase activity. The ULK1 complex is formed by ULK1, FIP200, ATG13, and ATG101 [49]. Under AA starvation conditions, mTORC1 is inactivated and dissociates from the ULK1 complex, recovering the kinase activity of the ULK1 complex. Activated ULK1 complex seems to be required for the phagophore formation, although LC3 is recruited independently of the ULK1 complex and Atg9 [50]. The intracellular AA pool normalizes 3–4 hr p.i., and mTORC1 localizes at the surface of the SCV and gets reactivated. Possibly, this reactivation of mTORC1 provides the escape of *Salmonella* from xenophagy in the late phase of the infection. The mechanisms, that lead to AA starvation, and how the bacteria can trigger AA normalization remain unknown and are important topics for further research.

#### Processes not included in the model

The third eat-me signal, besides galectin-8 and ubiquitin, is the lipid second messenger diacylglycerol (DAG) [51]. Early after infection, ∼45 min p.i., DAG accumulates to SCV. Most likely, the DAG-dependent pathway is independent of the ubiquitin-dependent xenophagy, since both ubiquitin and DAG-positive *Salmonella* have been observed, and inhibition of both pathways results in an additive effect on the recruitment of LC3 to the bacteria [51]. It is not clear whether DAG-positive *Salmonella* is degraded by autophagy or by a process called LC3-associated phagocytosis (LAP) [52, 53], or by both of them simultaneously. To distinguish between LAP and xenophagy, electron microscopic studies will be required in the future. We decided not to include the DAG-dependent pathway into the model, since it is not clear, whether DAG-positive *Salmonella* is degraded by autophagy or LAP. Additionally, to reduce the complexity of the model, we neglected a possible escape of *Salmonella* from the damaged SCV at later time points of infection.

Recently, a new autophagy receptor, Tax1-Binding Protein 1 (TAX1BP1), has been shown to play an important role in *Salmonella* xenophagy [54]. Contrary to the other autophagy receptors, OPTN, p62, and NDP52, the simultaneous depletion of TAX1BP1 and NDP52 leads to an additive effect on the restriction of *Salmonella* replication. It seems that TAX1BP1 and NDP52 have partially redundant functions in xenophagy and that TAX1BP1 can compensate the knockdown of NDP52. We decided not to include TAX1BP1 into the model, since it is not clear, whether TAX1BP1 alone is sufficient for capturing *Salmonella* to xenophagy or OPTN and p62 act along the same pathway.

### Petri net model of *Salmonella* xenophagy

**Fig 3:**
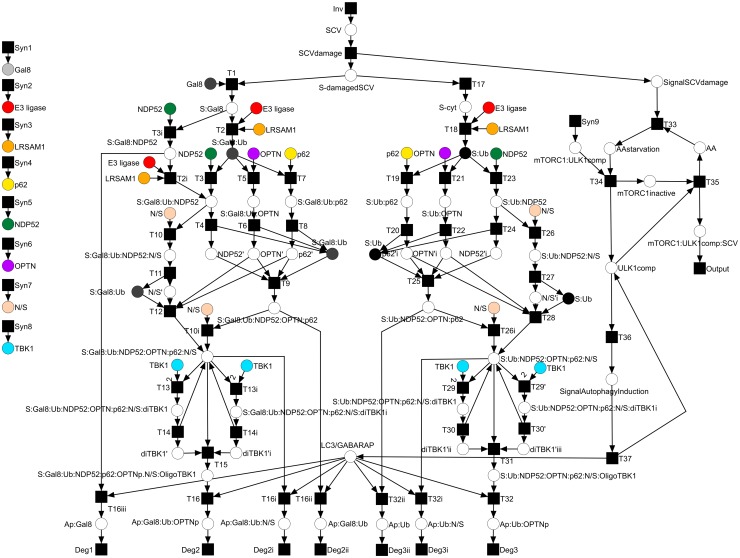
PN model of *Salmonella* xenophagy. The PN model comprises 61 places, including nine logical places represented by different colors, and 69 transitions connected by 184 arcs. All places and transitions, including a description and a reference, are listed in S1 and S2 Tables.

The PN describes the biological model shown in Fig 2. The model includes all known processes of *Salmonella* xenophagy in epithelial cells, which were biochemically proven and published, see S2 Table. We included a hypothetical model of TBK1 activation, suggesting a spatial and temporal regulation of this process, see detailed view in Fig 2. The resulting PN model of *Salmonella* xenophagy is available in S1 File. It comprises 61 places, including nine logical places, and 69 transitions connected by 184 arcs. A graphical representation of the PN model is depicted in Fig 3. All places and transitions, including their biological description, are listed in S1 and S2 Tables. Places can be proteins (e.g., *p62, NDP52, OPTN, TBK1*), macromolecular complexes (e.g., *S:Gal8, S:Ub:NDP52:OPTN:p62*), organisms (*S-cyt*), or even signals (e.g., *SignalSCVdamage, NDP52’*). The place *E3 ligase* represents an unknown E3 ubiquitin ligase. Ubiquitin and phosphate are not directly modeled as a place in the PN. Transitions can be binding processes (e.g., *T1, T2, T3*), escape from the SCV (*T17*), degradations (e.g., *Deg1, Deg2, Deg2i*), damage of the SCV (*SCVdamage*), or syntheses (*Syn1-9*). The PN has ten input and seven output transitions. Input transitions stand for the syntheses of proteins or protein complexes (*Syn1-9*) or the invasion of *Salmonella* to the cytosol (*SCVdamage*). Output transitions are degradations of captured *Salmonella* inside the autophagosome (e.g., *Deg1, Deg2, Deg2i*) or degradation of protein complexes (*Output*).

The temporal order of binding events in *Salmonella* xenophagy is still unknown. For instance, it is not known whether Nap1/Sintbad binds to NDP52 followed by a binding of the NDP52-Nap1/Sintbad complex to ubiquitinated *Salmonella* or whether NDP52 binds to ubiquitinated *Salmonella* followed by a binding of Nap1/Sintbad to NDP52-positive *Salmonella*. Likely, both temporal orders of binding events are possible. We decided to model one of these temporal orders as a typical system’s behavior. To model all possible orders is beyond the scope of this work and would unnecessarily increase the complexity of the model. The problem of combinatorial complexity emerges because the proteins can be modified and combined in various ways to form multicomponent complexes, reviewed by Hlavacek et al. [55].

#### Model analysis

We verified the PN model, using standard PN analysis techniques [17] to justify the biological reliability of the model. The PN is connected, covered by T-invariants, and each T-invariant has a meaningful biological interpretation.

The PN consists of 16 T-invariants listed in S3 Table, which describe biological subpathways at steady-state and reflect the basic dynamics of the system. Each of these T-invariants represents an alternative way of the autophagic capturing of *Salmonella*. An exemplary T-invariant is depicted in S2 Fig, which shows the ubiquitin-dependent xenophagy of *Salmonella* inside the cytosol, without Nap1/Sintbad binding and without OPTN phosphorylation. We can see that this process is connected with the AA level-dependent ULK1 activation. The existence of multiple ways of the autophagic capturing of *Salmonella* indicates the robustness of this key defense pathway against internal perturbations, such as mutations or silencing, and external perturbations, such as bacterial defense mechanisms.

The PN consists of 15 P-invariants listed in S4 Table. An exemplary P-invariant is shown in S3 Fig, which represents the conservation of NDP52. NDP52 is a component of 27 places/complexes, indicating its key role in *Salmonella* xenophagy. S4 Fig depicts a bar chart of the 15 P-invariants ordered according to their size, i.e. the number of places/complexes this substance is part of. As expected, *Salmonella* is present in most places of the net, precisely in 32 of the 61 places, and is therefore the most conserved biological entity in the model.

### *In silico* knockout analysis

To observe the effect of specific proteins on the network behavior, we performed *in silico* knockout experiments and compared the results with published experimental data. The aim was to check the biological credibility of the model. The model describes 16 different ways of capturing *Salmonella* for the xenophagy pathway. If we knockout each of the autophagy components, unknown E3 ubiquitin ligase, LRSAM1, galectin-8, p62, NDP52, OPTN, Nap1/Sintbad, or TBK1, all of these 16 ways or even a few could be affected.

**Fig 4:**
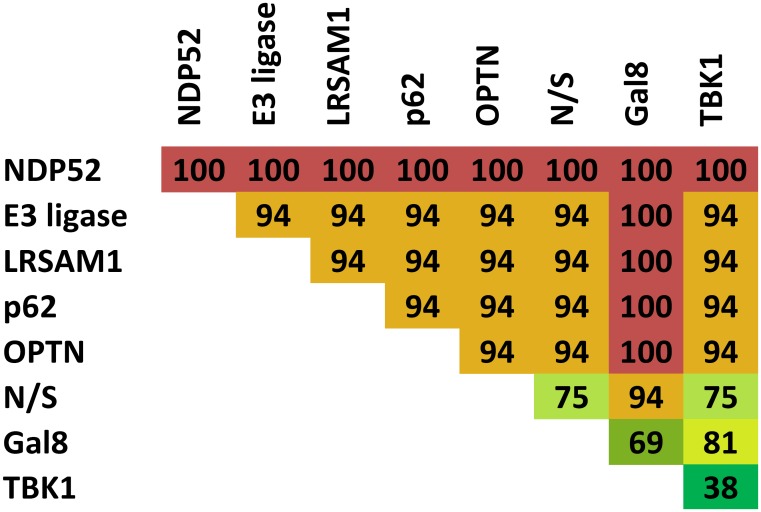
Sensitivity matrix of the *in silico* knockout analysis. The rows and the columns indicate the proteins knocked out. Thus, the diagonal of the matrix shows the results of the single knockouts and the other entries of the double knockouts. The numbers represent the percentage of T-invariants that are affected by a knockout. The colors indicate the impact on the xenophagy pathway (red = high, green = low).

S5 and S6 Tables list the knocked out proteins and their effect on *Salmonella* xenophagy measured in the percentage of affected T-invariants. In Fig 4, the results are visualized in the sensitivity matrix. The knockout analysis showed that only the knockout of NDP52 affects 100% of the T-invariants, resulting in a complete loss of function of both galectin-8 and ubiquitin-dependent xenophagy. None of the 16 T-invariants remained functional, which demonstrates the central role of NDP52 in *Salmonella* xenophagy. The knockout of the E3 ubiquitin ligase LRSAM1, other E3 ubiquitin ligases, and the autophagy receptors, p62 and OPTN, affected 94% of the T-invariants. Only the galectin-8-dependent xenophagy pathway remained functional. A double knockout of p62 and NDP52 showed a complete loss of function of the galectin-8 and ubiquitin-dependent xenophagy. This result is consistent with the double knockdown experiment of NDP52 and p62 in HeLa cells [38]. The knockdown of both autophagy receptors caused a reduction of LC3/GABARAP association to *Salmonella* to a similar extent than the knockdown of one receptor. A knockout of the kinase TBK1 would affect only 38% of the T-invariants. In the model, TBK1 is not absolutely required in xenophagy, but may have an enhancing effect on *Salmonella* xenophagy. Nevertheless, TBK1 siRNA-treated HeLa cells show similar *Salmonella* replication than NDP52 siRNA-treated cells [8, 9]. This may be due to a higher amount of fast-replicating, cytosolic bacteria caused by a reduced vacuolar integrity in TBK1-depleted cells [25].

**Fig 5:**
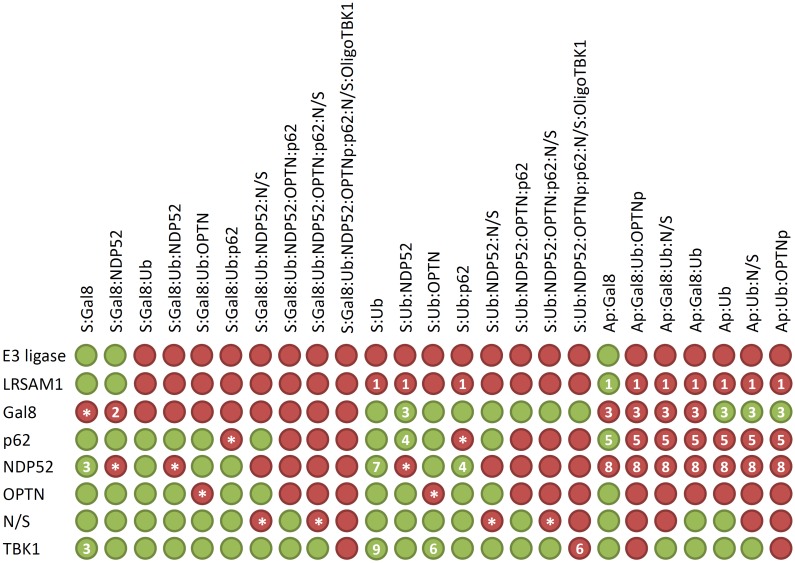
*In silico* knockout matrix. A row represents a protein knockout and a column the effect of the perturbation on a macromolecular *Salmonella* complex. A green entry indicates no effect and a red entry a negative effect, i.e., a reduced formation of a macromolecular complex. The numbers in some entries represent the reference literature of experimentally investigated effects: ^1^ Huett et al. 2012 [34]; ^2^ Thurston et al. 2012 [6], Li et al. 2013 [33]; ^3^ Thurston et al. 2012 [6]; ^4^ Cemma et al. 2011 [38]; ^5^ Zheng et al. 2009 [7], Cemma et al. 2011 [38]; ^6^ Wild et al. 2011 [9]; ^7^ Li et al. 2013 [33]; ^8^ Cemma et al. 2011 [38], Thurston et al. 2009 [8]; ^9^ Wild et al. 2011 [9], Radtke et al. 2007 [25]. The results marked by an asterisk are biologically obvious and need no further experimental investigation. The knockout of the seven places *Ap:Gal8, Ap:Gal8:Ub, Ap:Gal8:Ub:N/S, Ap:Ub, Ap:Ub:N/S, Ap:Gal8:Ub:OPTNp, Ap:Ub:OPTNp* is experimentally investigated, if the fraction of LC3/GABARAP-positive *Salmonella* has been observed in the experiments.

To avoid effects as described in Fig 1, we included additional output transitions to generate new T-invariants for the construction of the *in silico* knockout matrix. The modified PN model is available in S2 File. Fig 5 shows the resulting *in silico* knockout matrix. The rows of the matrix represent protein knockouts and the columns the effects of the perturbation on macromolecular *Salmonella* complexes. The matrix has 200 entries, 41 of those had been experimentally investigated in eight studies [6–9, 25, 33, 34, 38]. 12 entries are biologically obvious and need no further experimental investigation, e.g., the knockout of galectin-8 has a negative effect on the binding of galectin-8 to *Salmonella* inside the damaged SCV. The other 147 entries can be interpreted as hypotheses for future investigations. In the following, we describe the effect of some knocked out proteins (see Fig 5).

#### *In silico* knockout of LRSAM1

The *in silico* knockout of LRSAM1 resulted in a negative effect on the ubiquitination of *Salmonella* and on the binding of the autophagy receptors, NDP52, OPTN, and p62. Huett et al. reported a reduced association of ubiquitin, NDP52, and p62 with *Salmonella* in siLRSAM1-treated HeLa cells [34]. The reduced binding of OPTN to *Salmonella* by LRSAM1 knockdown remains to be examined in future studies.

#### *In silico* knockout of galectin-8

The *in silico* knockout of galectin-8 led to a reduced recruitment of NDP52 to galectin-8-positive *Salmonella* and had no effect on the association of NDP52 with ubiquitin-positive *Salmonella*. A reduced fraction of NDP52-positive *Salmonella* has been observed in HeLa cells knocked down for galectin-8 at 1 hr p.i. [8]. At 4 hr p.i., the recruitment of NDP52 to *Salmonella* seems not to be inhibited, which may be explained by a ubiquitin-dependent recruitment of NDP52 at later time points of the infection.

#### *In silico* knockout of p62

The knockout of p62 in the PN had a negative effect on the formation of autophagosomes. The transfection of HeLa cells with p62 siRNA has been shown to have a reduced percentage of LC3/GABARAP-positive *Salmonella* [7, 38]. Additionally, the *in silico* knockout of p62 revealed no effect on the binding of NDP52 or OPTN to *Salmonella*. The independent recruitment of p62 and NDP52 to *Salmonella* has been reported in HeLa cells [38], but there are no studies examining whether the p62 localization to *Salmonella* is independent of OPTN localization.

#### *In silico* knockout of NDP52

The *in silico* knockout of NDP52 led to no negative effect on the ubiquitination of *Salmonella*. Thurston et al. observed even an increase of ubiquitinated *Salmonella* in NDP52-depleted HeLa cells [8], which may be caused by a higher fraction of cytosolic bacteria due to an inoperable autophagic degradation. Furthermore, the *in silico* knockout of NDP52 had no effect on the binding of p62 and OPTN to bacteria. The knockdown of NDP52 in HeLa cells has been shown to have no influence on the percentage of p62-positive *Salmonella* [38]. It has to be studied whether the recruitment of OPTN to *Salmonella* is also independent of NDP52. Moreover, the knockout of NDP52 in the model showed a negative effect on the formation of autophagosomes. The inhibition of LC3 recruitment to bacteria has been detected in NDP52-depleted HeLa cells in two studies [8, 38].

#### *In silico* knockout of OPTN and Nap1/Sintbad

We could not find knockout or knockdown experiments in the literature that had observed the influence of OPTN and Nap1/Sintbad on any protein complex included in the PN. The analysis of the PN model hypothesized that the knockout of OPTN had no influence on the ubiquitination and the recruitment of NDP52, p62, and Nap1/Sintbad to *Salmonella*. This hypothesis has to be experimentally validated in further studies.

#### *In silico* knockout of TBK1

The knockout of TBK1 in the PN showed no negative effect on the ubiquitination of *Salmonella*, the binding of the autophagy receptors, and the binding of Nap1/Sintbad to *Salmonella*. In *Tbk1^−/−^* MEFs, an even higher amount of ubiquitinated *Salmonella* has been observed [25]. The increased amount of ubiquitinated *Salmonella* in *Tbk1^−/−^* MEFs can be explained by another function of TBK1, besides OPTN phosphorylation. TBK1 has been reported to maintain the integrity of the SCV [25]. So, the knockout of TBK1 leads to a higher amount of bacteria inside the cytosol, which results in a higher fraction of ubiquitinated *Salmonella*. We decided to exclude the process of maintaining the integrity of the SCV by TBK1 from the model, because it is not known how TBK1 can influence the integrity of the SCV. In contrast to *Tbk1^−/−^* MEFs, a reduced association of ubiquitin with *Salmonella sifA^−^* in siTBK1-treated HeLa cells has been reported [9]. *Salmonella sifA^−^* is a *Salmonella* mutant that remains predominantly in the cytosol. It has to be tested, whether the knockout or knockdown of TBK1 in *Salmonella*-infected HeLa cells has no influence on the ubiquitination of the bacteria as predicted by the model. A higher fraction of ubiquitinated *Salmonella* in siTBK1-treated cells can also be explained by a higher fraction of cytosolic bacteria caused by the loss of vacuolar integrity. The *in silico* knockout of TBK1 had a negative effect on OPTN phosphorylation. The knockdown of TBK1 in *Salmonella sifA^−^*-infected HeLa has been shown to result in a reduced fraction of phosphorylated OPTN (pS177) [9].

### Conclusion

We constructed a mathematical model, which provides a compilation of currently available experimental findings of *Salmonella* xenophagy in a verified and consistent network topology of the elementary molecular processes. The PN model combines the molecular processes of ubiquitination, binding of the autophagy receptors, regulatory processes, like nutrient-dependent regulation of xenophagy, TBK1-dependent activation of the autophagy receptor, OPTN, and galectin-8-dependent xenophagy. Modeling the unknown process of TBK1 activation in HeLa cells, we proposed a new hypothetical model of TBK1 activation. We assume a spatial and temporal regulation of this process.

We checked the model structure for consistencies and correctness. We found 16 basic functional modules, which describe different pathways of the autophagic capturing of *Salmonella* and reflect the basic dynamics of the system. We introduced a new concept of *in silico* knockout analyses based on T-Invariants and visualized the results as an *in silico* knockout matrix. The model correctly reproduces the experimental results published so far, ensuring the biological credibility of the model. Based on the *in silico* knockout analyses, we postulate hypotheses for future investigations. Due to the fact that xenophagy was discovered recently and the exact molecular details are still unknown, the proposed model is probably not complete and will be extended by new insights. If quantitative data become available, the PN model can be easily extended to model the quantitative system’s behavior.

## Supporting Information

S1 File File of the *Salmonella* xenophagy model. (MLPROJECT)

S2 File File of the modified *Salmonella* xenophagy model for the *in silico* knockout analysis. (MLPROJECT)

S1 Fig Iterative modeling process. The first step is an extensive literature research. Then, the model is constructed and analyzed. The last step is the model verification, which can reveal inconsistencies. These steps have to be repeated until the model has no inconsistencies anymore, and all relevant experimental findings are included.
(TIF)

S2 Fig Example of a T-invariant. The T-invariant (ID TI12, red colored) contains 24 transitions, which represent the ubiquitin-dependent xenophagy of *Salmonella* inside the cytosol.
(TIF)

S3 Fig Example of a P-invariant. The P-invariant (ID PI7, red colored) contains 27 places, which represent the conservation of NDP52 in the system. NDP52 is a component of 44% of the places what demonstrates its critical role in *Salmonella* xenophagy.
(TIF)

S4 Fig Bar chart of the P-invariants. All P-invariants are listed according to their size. For example, *Salmonella* is the most conserved substance, as expected. We use the same color code as in Fig 5.
(TIF)

S1 Table Places (substances) of the Petri net. (PDF)

S2 Table Transitions (reactions) of the Petri net. (PDF)

S3 Table T-invariants of the Petri net. (PDF)

S4 Table P-invariants of the Petri net. (PDF)

S5 Table Single knockouts and their impact on *Salmonella* xenophagy. (PDF)

S6 Table Double knockouts and their impact on *Salmonella* xenophagy. (PDF)
