# Supplementary figures and images for: In Silico Knockout Studies of Xenophagic Capturing of Salmonella
Source: PLoS Comput Biol. 2016 Dec 1;12(12):e1005200. doi: 10.1371/journal.pcbi.1005200 (PMC5131900; doi:10.1371/journal.pcbi.1005200)

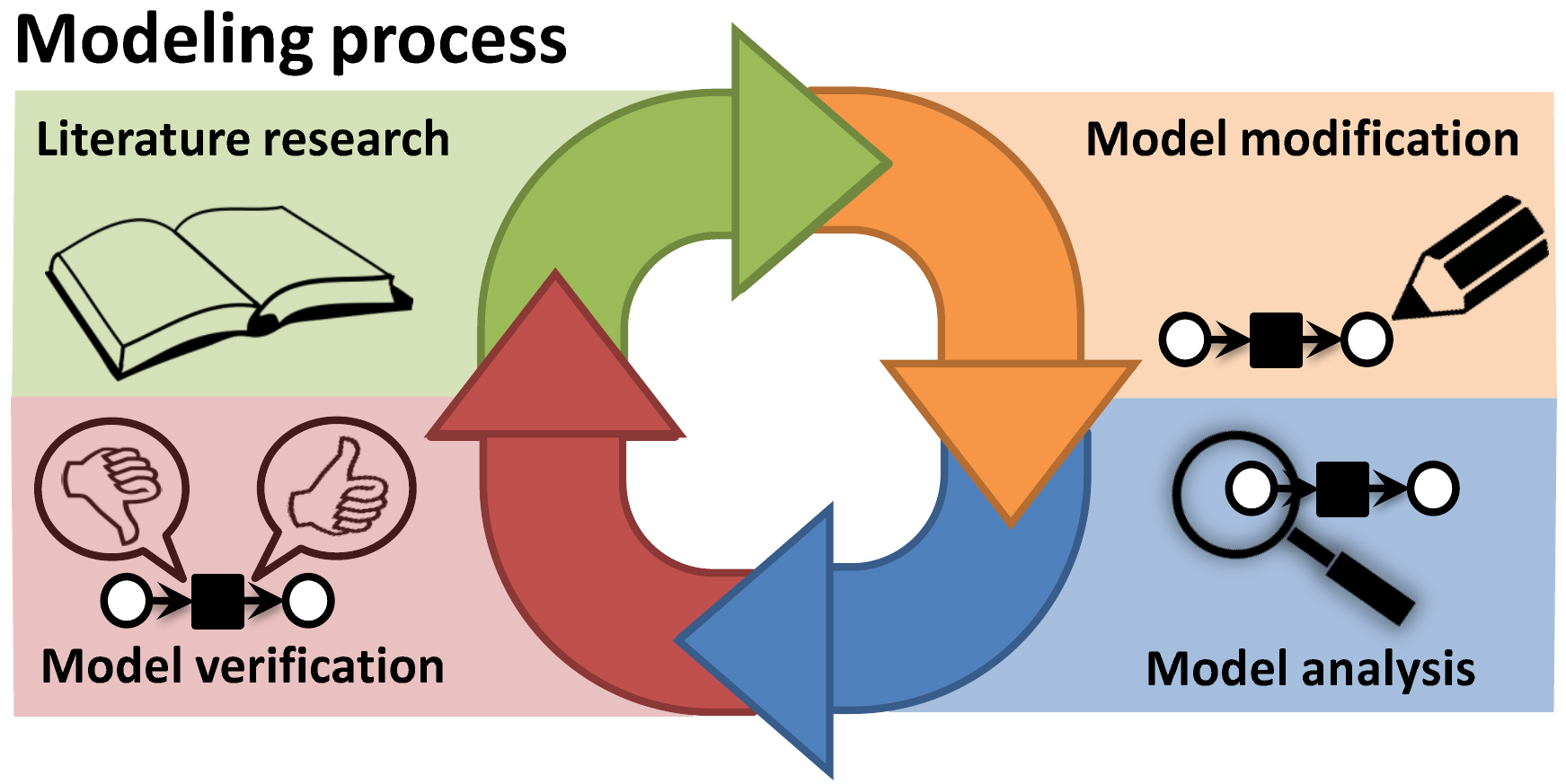

Supplement: S1 Fig — The first step is an extensive literature research. Then, the model is constructed and analyzed. The last step is the model verification, which can reveal inconsistencies. These steps have to be repeated until the model has no inconsistencies anymore, and all relevant experimental findings are included. (TIF) [file pcbi.1005200.s003.tif]

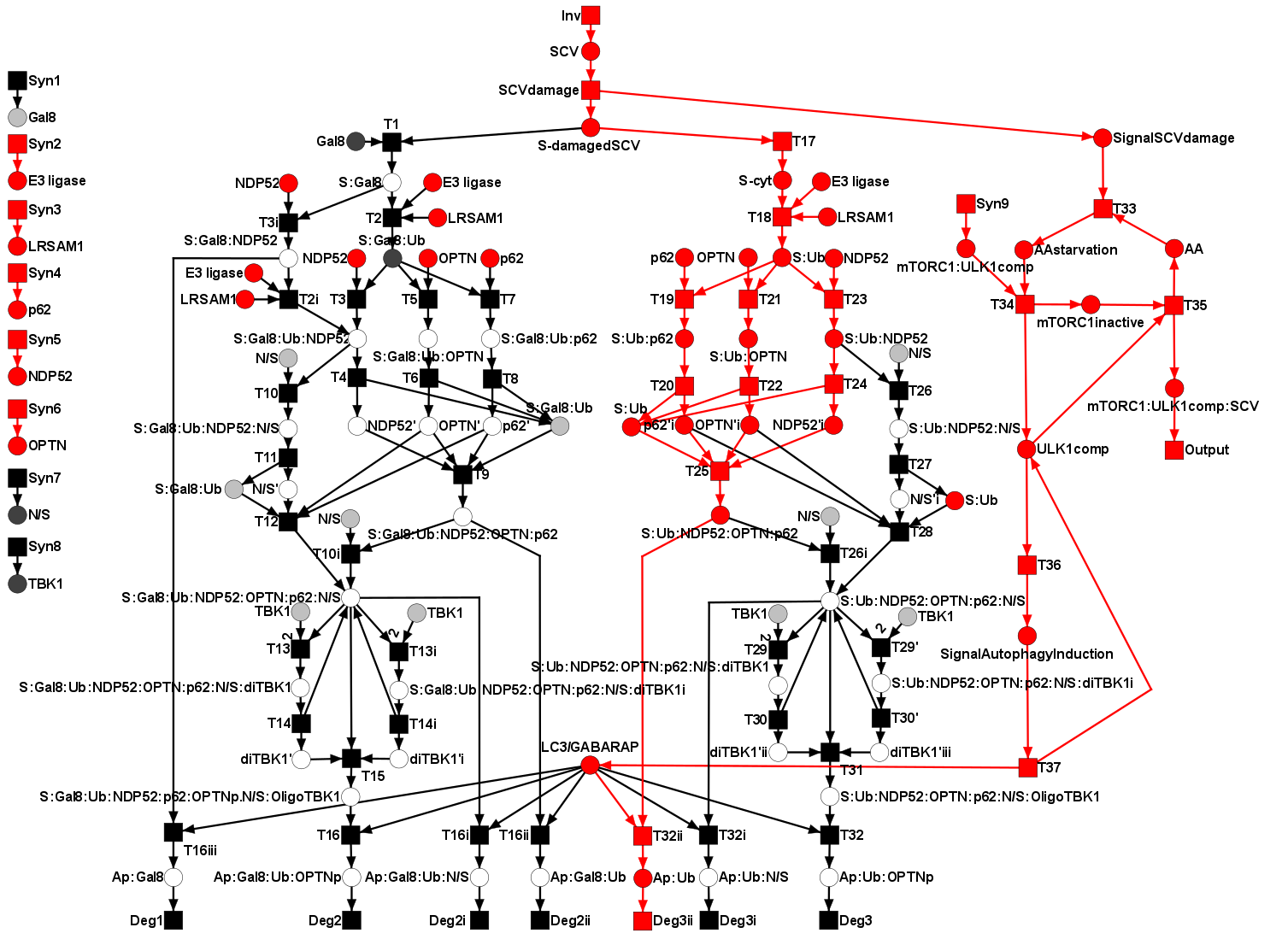

Supplement: S2 Fig — The T-invariant (ID TI12, red colored) contains 24 transitions, which represent the ubiquitin-dependent xenophagy of Salmonella inside the cytosol. (TIF) [file pcbi.1005200.s004.tif]

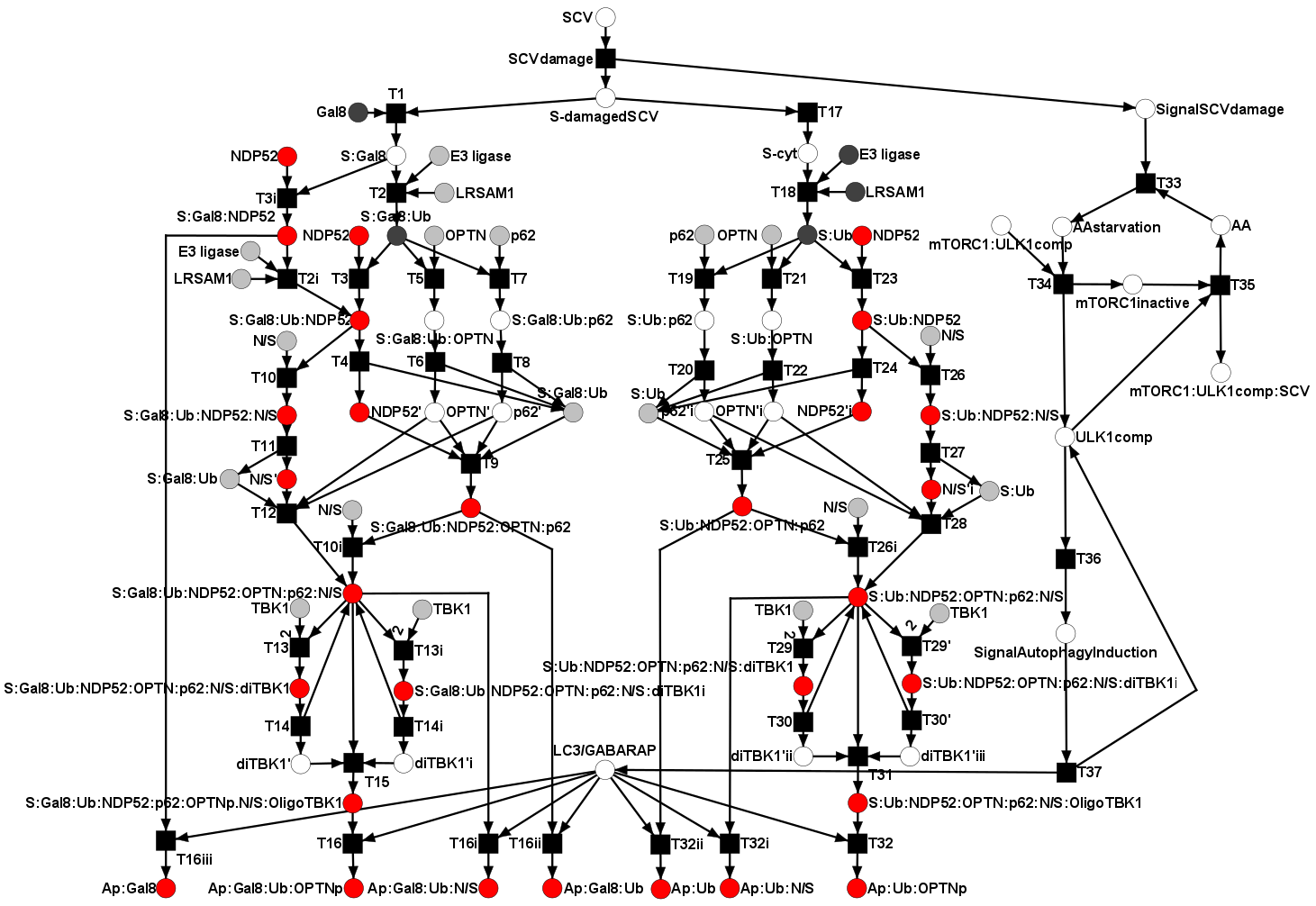

Supplement: S3 Fig — The P-invariant (ID PI7, red colored) contains 27 places, which represent the conservation of NDP52 in the system. NDP52 is a component of 44% of the places what demonstrates its critical role in Salmonella xenophagy. (TIF) [file pcbi.1005200.s005.tif]

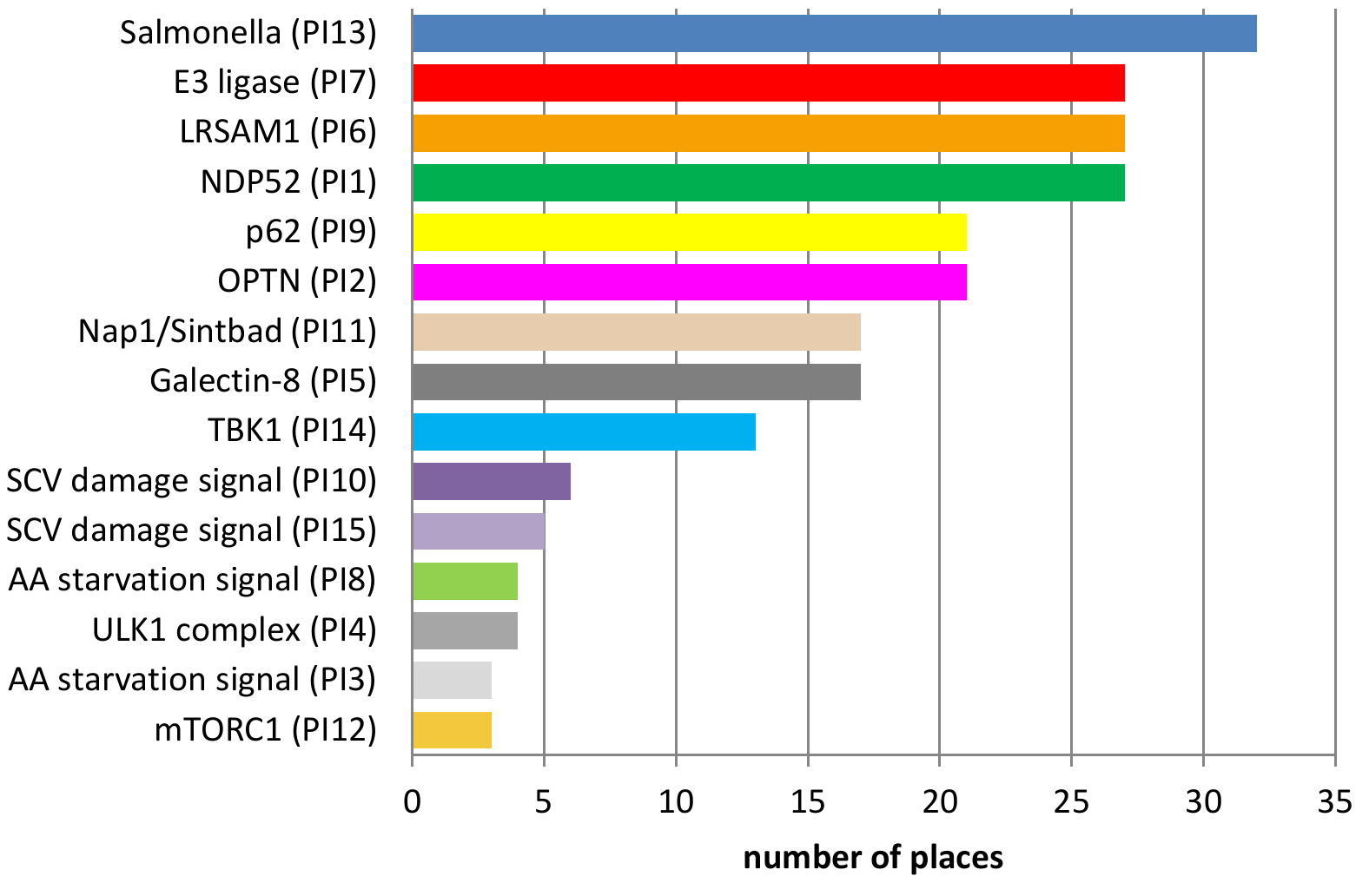

Supplement: S4 Fig — All P-invariants are listed according to their size. For example, Salmonella is the most conserved substance, as expected. We use the same color code as in Fig 5. (TIF) [file pcbi.1005200.s006.tif]
