# Supplementary material for: In Silico Knockout Studies of Xenophagic Capturing of Salmonella
Source: PLoS Comput Biol. 2016 Dec 1;12(12):e1005200. doi: 10.1371/journal.pcbi.1005200 (PMC5131900; doi:10.1371/journal.pcbi.1005200)
Supplement: S1 Table — (PDF) [file pcbi.1005200.s007.pdf]

S1 Table: Places (substances) of the Petri net.

**Place (#60)**	**Description**
SCV	*Salmonella* containing vacuole
S-damagedSCV	*Salmonella* inside a damaged SCV
S-cyt	*Salmonella* inside the cytosol
Gal8 (2 logical places)	Galectin-8
LRSAM1 (4 logical places)	Ubiquitin ligase LRSAM1
E3 ligase (4 logical places)	unknown E3 ligase or E3 ligases
NDP52 (4 logical places)	Autophagy receptor NDP52
NDP52'	Technical place type 0 of NDP52-positive *Salmonella*
NDP52'i	Technical place type i of NDP52-positive *Salmonella*
OPTN (3 logical places)	Autophagy receptor OPTN
OPTN'	Technical place type 0 of OPTN-positive *Salmonella*
OPTN'i	Technical place type i of OPTN-positive *Salmonella*
p62 (3 logical places)	Autophagy receptor p62
p62'	Technical place type 0 of p62-positive *Salmonella*
p62'i	Technical place type i of p62-positive *Salmonella*
TBK1 (5 logical places)	Kinase TBK1
diTBK1'	Technical place type 0 of dimeric TBK1-positive *Salmonella*
diTBK1'i	Technical place type i of dimeric TBK1-positive *Salmonella*
diTBK1'ii	Technical place type ii of dimeric TBK1-positive *Salmonella*
diTBK1'iii	Technical place type iii of dimeric TBK1-positive *Salmonella*
N/S (5 logical places)	Nap1 or Sintbad
N/S'	Technical place type 0 of Nap1/Sintbad-positive *Salmonella*
N/S'i	Technical place type i of Nap1/Sintbad-positive *Salmonella*
S:Gal8	Complex of *Salmonella* and galectin-8
S:Gal8:NDP52	Complex of *Salmonella* , galectin-8, and NDP52
S:Gal8:Ub (3 logical places)	Complex of *Salmonella* , galectin-8, and ubiquitin
S:Gal8:Ub:p62	Complex of *Salmonella* , galectin-8, ubiquitin, and p62

S:Gal8:Ub:OPTN	Complex of *Salmonella* , galectin-8, ubiquitin, and OPTN
S:Gal8:Ub:NDP52	Complex of *Salmonella* , galectin-8, ubiquitin, and NDP52
S:Gal8:Ub:NDP52:N/S	Complex of *Salmonella* , galectin-8, ubiquitin, NDP52, and Nap1/Sintbad
S:Gal8:Ub:NDP52:OPTN:p62	Complex of *Salmonella* , galectin-8, ubiquitin, NDP52, OPTN, and p62
S:Gal8:Ub:NDP52:OPTN:p62:N/S	Complex of *Salmonella* , galectin-8, ubiquitin, NDP52, OPTN, p62, and Nap1/Sintbad
S:Gal8:Ub:NDP52:OPTN:p62:N/S:diTBK1	Complex of *Salmonella* , galectin-8, ubiquitin, NDP52, OPTN, p62, Nap1/Sintbad, and dimeric TBK1 type 0
S:Gal8:Ub:NDP52:OPTN:p62:N/S:diTBK1i	Complex of *Salmonella* , galectin-8, ubiquitin, NDP52, OPTN, p62, Nap1/Sintbad, and dimeric TBK1 type i
S:Gal8:Ub:NDP52:OPTNp:p62:N/S:OligoTBK1	Complex of *Salmonella* , galectin-8, ubiquitin, NDP52, phosphorylated OPTN, p62, Nap1/Sintbad, and oligomerized TBK1 dimers
S:Ub	Complex of *Salmonella* and ubiquitin
S:Ub:p62	Complex of *Salmonella* , ubiquitin, and p62
S:Ub:OPTN	Complex of *Salmonella* , ubiquitin, and OPTN
S:Ub:NDP52	Complex of *Salmonella* , ubiquitin, and NDP52
S:Ub:NDP52:N/S	Complex of *Salmonella* , ubiquitin, NDP52, and Nap1/Sintbad
S:Ub:NDP52:OPTN:p62	Complex of *Salmonella* , ubiquitin, NDP52, OPTN, and p62
S:Ub:NDP52:OPTN:p62:N/S	Complex of *Salmonella* , ubiquitin, NDP52, OPTN, p62, and Nap1/Sintbad
S:Ub:NDP52:OPTN:p62:N/S:diTBK1	Complex of *Salmonella* , ubiquitin, NDP52, OPTN, p62, Nap1/Sintbad, and dimeric TBK1
S:Ub:NDP52:OPTN:p62:N/S:diTBK1i	Complex of *Salmonella* , ubiquitin, NDP52, OPTN, p62, Nap1/Sintbad, and dimeric TBK1
S:Ub:NDP52:OPTNp:p62:N/S:OligoTBK1	Complex of *Salmonella* , ubiquitin, NDP52, phosphorylated OPTN, p62, Nap1/Sintbad, and oligomerized TBK1 dimers
LC3/GABARAP	LC3/GABARAP
SignalSCVdamage	Technical place of the SCV damage
AA	Normal amino acid level
AAstarvation	Amino acid starvation

mTORC1:ULK1comp	Complex of active mTORC1 and the inactivated ULK1 complex
mTORC1:ULK1comp:SCV	Complex of active mTORC1 on the SCV and the inactivated ULK1 complex
mTORC1inactive	inactivated mTORC1 (formed by mTOR, Raptor, and mLST8/G*beta*L)
ULK1comp	activated ULK1 complex (formed by ULK1, FIP200, ATG13, and ATG101)
SignalAutophagyInduction	Technical place of the xenophagy induction
Ap:Gal8	Autophagosome, including galectin-8-positive *Salmonella*
Ap:Gal8:Ub:N/S	Autophagosome, including galectin-8-positive, ubiquitinated *Salmonella*, and Nap1/Sintbad
Ap:Gal8:Ub	Autophagosome, including galectin-8-positive, ubiquitinated *Salmonella*
Ap:Ub	Autophagosome, including ubiquitinated *Salmonella*
Ap:Ub:N/S	Autophagosome, including ubiquitinated *Salmonella*, and Nap1/Sintbad
Ap:Gal8:Ub:OPTNp	Autophagosome, including galectin-8-positive, ubiquitinated *Salmonella*, and phosphorylation of OPTN
Ap:Ub:OPTNp	Autophagosome, including ubiquitinated *Salmonella*, and phosphorylation of OPTN
