# Supplementary material for: In Silico Knockout Studies of Xenophagic Capturing of Salmonella
Source: PLoS Comput Biol. 2016 Dec 1;12(12):e1005200. doi: 10.1371/journal.pcbi.1005200 (PMC5131900; doi:10.1371/journal.pcbi.1005200)
Supplement: S2 Table — (PDF) [file pcbi.1005200.s008.pdf]

S2 Table: Transitions (reactions) of the Petri net.

Transition	Description	Reference
Inv	Invasion of *Salmonella* into the epithelial cell	Gomes et al. 2014, Stolz et al. 2014
SCVdamage	Disruption of the SCV	Beuzon et al. 2002, Brumell et al. 2002
T1	Binding of galectin-8 to host glycans exposed on SCV	Thurston et al. 2012
T2	Binding of LRSAM1 and other unknown E3 ligases to galectin-8-positive *Salmonella* and ubiquitination of *Salmonella*	Huett et al. 2012, Ng et al. 2011, Perrin et al. 2004
T2i	Binding of LRSAM1 and other unknown E3 ligases to galectin-8 and NDP52-positive *Salmonella* and ubiquitination of *Salmonella*	Huett et al. 2012, Ng et al. 2011, Perrin et al. 2004
T3	Recruitment of NDP52 to galectin-8 and/or ubiquitin-positive *Salmonella*	Thurston et al. 2009, Thurston et al. 2012, Li et al. 2013
T3i	Binding of NDP52 to galectin-8	Thurston et al. 2012, Li et al. 2013
T4	Complex formation of NDP52 to galectin-8 and/or ubiquitin-positive *Salmonella*	Thurston et al. 2009, Thurston et al. 2012, Li et al. 2013
T5	Recruitment of OPTN to ubiquitin and galectin-8-positive *Salmonella*	Wild et al. 2011
T6	Complex formation of OPTN to ubiquitin and galectin-8-positive *Salmonella*	Wild et al. 2011
T7	Recruitment of p62 to ubiquitin and galectin-8-positive *Salmonella*	Zheng et al. 2009
T8	Complex formation of p62 to ubiquitin and galectin-8-positive *Salmonella*	Zheng et al. 2009
T9	Binding of the three autophagy receptors, p62, OPTN, and NDP52, to galectin-8 and ubiquitin-positive *Salmonella*	Zheng et al. 2009, Thurston et al. 2009, Wild et al. 2011 Thurston et al. 2012, Li et al. 2013
T10	Recruitment of Nap1/Sintbad to NDP52-positive *Salmonella*	Thurston et al. 2009
T10i	Binding of Nap1/Sintbad to NDP52, p62 and OPTN-positive *Salmonella* complex	Thurston et al. 2009

T11	Complex formation of Nap1/Sintbad to NDP52-positive *Salmonella*	Thurston et al. 2009
T12	Binding of the autophagy receptors, p62 and OPTN, to galectin-8 and ubiquitin-positive *Salmonella* complex, including NDP52-Nap1/Sintbad complex	Zheng et al. 2009, Wild et al. 2011
T13	Recruitment of dimeric TBK1 type 0 to OPTN and NDP52 via Nap1/Sintbad	Ryzhakov et al. 2007, Thurston et al. 2009, Tu et al. 2013, Larabi et al. 2013
T13i	Recruitment of dimeric TBK1 type i to OPTN and NDP52 via Nap1/Sintbad	Ryzhakov et al. 2007, Thurston et al. 2009, Tu et al. 2013, Larabi et al. 2013
T14	Complex formation of dimeric TBK1 type 0 to OPTN and NDP52 via Nap1/Sintbad	Ryzhakov et al. 2007, Thurston et al. 2009, Tu et al. 2013, Larabi et al. 2013
T14i	Complex formation of dimeric TBK1 type i to OPTN and NDP52 via Nap1/Sintbad	Ryzhakov et al. 2007, Thurston et al. 2009, Tu et al. 2013, Larabi et al. 2013
T15	Oligomerization of TBK1 leads to autoactivation and phosphorylation of OPTN	Fujita et al. 2003, Ma et al.2012, Shu et al.2013, Larabi et al. 2013, Gleason et al. 2011
T16	Binding of the galectin-8-positive, ubiquitinated *Salmonella* complex including phosphorylated OPTN to LC3/GABARAP leads to autophagosome formation	Birmingham et al. 2006, Morton et al. 2008, Wild et al. 2011, Rogov et al. 2013
T16i	Binding of the galectin-8-positive, ubiquitinated *Salmonella* complex including the NDP52-Nap1/Sintbad complex to LC3/GABARAP leads to autophagosome formation	Birmingham et al. 2006
T16ii	Binding of the galectin-8-positive, ubiquitinated *Salmonella* complex to LC3/GABARAP leads to autophagosome formation	Birmingham et al. 2006
T16iii	Binding of the galectin-8-positive *Salmonella* complex to LC3/GABARAP leads to autophagosome formation	Birmingham et al. 2006, Thurston et al. 2012, Li et al. 2013
T17	*Salmonella* escape from the damaged SCV	Birmingham et al. 2006, Yu et al. 2014

T18	Binding of LRSAM1 to cytosolic *Salmonella* and ubiquitination of *Salmonella*	Huett et al. 2012, Ng et al. 2011, Perrin et al. 2004
T19	Recruitment of p62 to ubiquitin-positive *Salmonella*	Zheng et al. 2009
T20	Complex formation of p62 to ubiquitin-positive *Salmonella*	Zheng et al. 2009
T21	Recruitment of OPTN to ubiquitin-positive *Salmonella*	Wild et al. 2011
T22	Complex formation of OPTN to ubiquitin-positive *Salmonella*	Wild et al. 2011
T23	Recruitment of NDP52 to ubiquitin-positive *Salmonella*	Thurston et al. 2009
T24	Complex formation of NDP52 to ubiquitin-positive *Salmonella*	Thurston et al. 2009
T25	Binding of the three autophagy receptors, p62, OPTN, and NDP52, to ubiquitin-positive *Salmonella*	Zheng et al. 2009, Thurston et al. 2009, Wild et al. 2011 Thurston et al. 2012, Li et al. 2013
T26	Recruitment of Nap1/Sintbad to NDP52-positive cytosolic *Salmonella*	Thurston et al. 2009
T26i	Binding of Nap1/Sintbad to NDP52-positive cytosolic *Salmonella*	Thurston et al. 2009
T27	Complex formation of Nap1/Sintbad to NDP52-positive cytosolic *Salmonella*	Thurston et al. 2009
T28	Binding of the autophagy receptors, p62 and OPTN, to ubiquitin-positive *Salmonella* complex, including NDP52-Nap1/Sintbad complex	Zheng et al. 2009, Wild et al. 2011
T29	Recruitment of dimeric TBK1 type 0 to OPTN and NDP52-positive cytosolic *Salmonella* via Nap1/Sintbad	Ryzhakov et al. 2007, Thurston et al. 2009, Tu et al. 2013, Larabi et al. 2013
T29'	Recruitment of dimeric TBK1 type i to OPTN and NDP52-positive cytosolic *Salmonella* via Nap1/Sintbad	Ryzhakov et al. 2007, Thurston et al. 2009, Tu et al. 2013, Larabi et al. 2013
T30	Complex formation of dimeric TBK1 type 0 to OPTN and NDP52-positive cytosolic *Salmonella* via Nap1/Sintbad	Ryzhakov et al. 2007, Thurston et al. 2009, Tu et al. 2013, Larabi et al. 2013

T30'	Complex formation of dimeric TBK1 type i to OPTN and NDP52-positive cytosolic *Salmonella* via Nap1/Sintbad	Ryzhakov et al. 2007, Thurston et al. 2009, Tu et al. 2013, Larabi et al. 2013
T31	Oligomerization of TBK1 leads to autoactivation and phosphorylation of OPTN at cytosolic *Salmonella*	Fujita et al. 2003, Ma et al.2012, Shu et al.2013, Larabi et al. 2013, Gleason et al. 2011
T32	Binding of ubiquitinated *Salmonella* complex including phosphorylated OPTN to LC3/GABARAP leads to autophagosome formation	Morton et al. 2008, Wild et al. 2011, Rogov et al. 2013
T32i	Binding of ubiquitinated *Salmonella* complex including the NDP52-Nap1/Sintbad complex to LC3/GABARAP leads to autophagosome formation	Birmingham et al. 2006
T32ii	Binding of ubiquitinated *Salmonella* complex to LC3/GABARAP leads to autophagosome formation	Birmingham et al. 2006
T33	AA starvation is triggered by SCV damage	Tattoli et al. 2012_1, Tattoli et al. 2012_2
T34	AA starvation leads to the inactivation of mTORC1 and its dissociation of the ULK1 complex	Tattoli et al. 2012_1, Tattoli et al. 2012_2, Mizushima 2010
T35	Normalization of the AA level leads to mTORC1 reactivation and mTORC1 localizes to the surface of the SCV, resulting in inactivation of ULK1 complex	Tattoli et al. 2012_1, Tattoli et al. 2012_2, Mizushima 2010
T36	Activated ULK1 complex induces xenophagy	Mizushima 2010
T37	Activated ULK1 complex induces formation of phagophores	Mizushima 2010
Deg1	Degradation of the autophagosome through fusion with lysosome (output transition)	Gomes et al. 2014, Stolz et al. 2014
Deg2	Degradation of the autophagosome through fusion with lysosome (output transition)	Gomes et al. 2014, Stolz et al. 2014
Deg2i	Degradation of the autophagosome through fusion with lysosome (output transition)	Gomes et al. 2014, Stolz et al. 2014
Deg2ii	Degradation of the autophagosome through fusion with lysosome (output transition)	Gomes et al. 2014, Stolz et al. 2014

Deg3	Degradation of the autophagosome through fusion with lysosome (output transition)	Gomes et al. 2014, Stolz et al. 2014
Deg3i	Degradation of the autophagosome through fusion with lysosome (output transition)	Gomes et al. 2014, Stolz et al. 2014
Deg3ii	Degradation of the autophagosome through fusion with lysosome (output reaction)	Gomes et al. 2014, Stolz et al. 2014
Output	Technical output transition (output transition)	
Syn1	Synthesis of galectin-8 (input transition)	
Syn2	Synthesis of ubiquitin (input transition)	
Syn3	Synthesis of LRSAM1 or other E3-ligases (input transition)	
Syn4	Synthesis of p62 (input transition)	
Syn5	Synthesis of NDP52 (input transition)	
Syn6	Synthesis of OPTN (input transition)	
Syn7	Synthesis of TLR4 (input reaction)	
Syn8	Synthesis of TBK1 (input transition)	
Syn9	Complex formation of active mTORC1 and the inactivated ULK1 complex (input transition)	Mizushima 2010
