# Supplementary material for: In Silico Knockout Studies of Xenophagic Capturing of Salmonella
Source: PLoS Comput Biol. 2016 Dec 1;12(12):e1005200. doi: 10.1371/journal.pcbi.1005200 (PMC5131900; doi:10.1371/journal.pcbi.1005200)
Supplement: S3 Table — (PDF) [file pcbi.1005200.s009.pdf]

S3 Table: T-invariants of the Petri net.

T-invariant ID (Size)	Transitions (Reactions)	Biological Meaning
TI1 (15)	Inv, T1, SCVdamage, T16iii, Deg1, T35, Syn1, T3i, T33, T36, Syn5, T37, T34, Syn9, Output	Galectin-8-dependent xenophagy of *Salmonella* inside a damaged SCV.
TI2 (37)	Inv, SCVdamage, T1, Syn1, T35, T3i, T33, T6, T8, T36, Syn7, Syn5, Syn6, T2i, T34, T7, T5, T13, Deg2, T16, Syn4, Syn3, T14, T13i, Syn2, T37, T14i, T15, 4*Syn8, T10, Syn9, T11, T12, Output	Galectin-8 and ubiquitin-dependent xenophagy of *Salmonella* inside a damaged SCV, including phosphorylation of OPTN.
TI3 (37)	Inv, SCVdamage, T1, T9, Syn1, T35, T4, T3i, T33, T6, T8, T36, Syn7, Syn5, Syn6, T2i, T34, T7, T5, T10i, T13, Deg2, T16, Syn4, Syn3, T14, T13i, Syn2, T37, T14i, T15, 4*Syn8, Syn9, Output	Galectin-8 and ubiquitin-dependent xenophagy of *Salmonella* inside a damaged SCV, including phosphorylation of OPTN.
TI4 (37)	Inv, SCVdamage, T1, T9, Syn1, T35, T4, T33, T6, T8, T36, Syn7, Syn5, Syn6, T34, T2, T3, T7, T5, T10i, T13, Deg2, T16, Syn4, Syn3, T14, T13i, Syn2, T37, T14i, T15, 4*Syn8, Syn9, Output	Galectin-8 and ubiquitin-dependent xenophagy of *Salmonella* inside a damaged SCV, including phosphorylation of OPTN.
TI5 (37)	Inv, SCVdamage, T1, Syn1, T35, T33, T6, T8, T36, Syn7, Syn5, Syn6, T34, T2, T3, T7, T5, T13, Deg2, T16, Syn4, Syn3, T14, T13i, Syn2, T37, T14i, T15, 4*Syn8, T10, Syn9, T11, T12, Output	Galectin-8 and ubiquitin-dependent xenophagy of *Salmonella* inside a damaged SCV, including phosphorylation of OPTN.
TI6 (28)	Inv, SCVdamage, T1, T9, Syn1, T35, T4, T3i, T33, T6, T8, T36, Syn7, Syn5, Syn6, T2i, T34, T7, T5, T10i, Syn4, Syn3, Syn2, T37, Syn9, T16i, Deg2i, Output	Galectin-8 and ubiquitin-dependent xenophagy of *Salmonella* inside a damaged SCV, including Nap1/Sintbad binding to NDP52.
TI7 (28)	Inv, SCVdamage, T1, Syn1, T35, T3i, T33, T6, T8, T36, Syn7, Syn5, Syn6, T2i, T34, T7, T5, Syn4, Syn3, Syn2, T37, T10, Syn9, T11, T16i, Deg2i, T12, Output	Galectin-8 and ubiquitin-dependent xenophagy of *Salmonella* inside a damaged SCV, including Nap1/Sintbad binding to NDP52.

TI8 (28)	Inv, SCVdamage, T1, Syn1, T35, T33, T6, T8, T36, Syn7, Syn5, Syn6, T34, T2, T3, T7, T5, Syn4, Syn3, Syn2, T37, T10, Syn9, T11, T16i, Deg2i, T12, Output	Galectin-8 and ubiquitin-dependent xenophagy of *Salmonella* inside a damaged SCV, including Nap1/Sintbad binding to NDP52.
TI9 (28)	Inv, SCVdamage, T1, T9, Syn1, T35, T4, T33, T6, T8, T36, Syn7, Syn5, Syn6, T34, T2, T3, T7, T5, T10i, Syn4, Syn3, Syn2, T37, Syn9, T16i, Deg2i, Output	Galectin-8 and ubiquitin-dependent xenophagy of *Salmonella* inside a damaged SCV, including Nap1/Sintbad binding to NDP52.
TI10 (26)	Inv, SCVdamage, T1, T9, Syn1, T35, T4, T33, T6, T8, T36, Syn5, Syn6, T34, T2, T3, T7, T5, Syn4, Syn3, T37, Syn2, T16ii, Syn9, Deg2ii, Output	Galectin-8 and ubiquitin-dependent xenophagy of *Salmonella* inside a damaged SCV.
TI11 (26)	Inv, SCVdamage, T1, T9, Syn1, T35, T4, T3i, T33, T6, T8, T36, Syn5, Syn6, T2i, T34, T7, T5, Syn4, Syn3, T37, Syn2, T16ii, Syn9, Deg2ii, Output	Galectin-8 and ubiquitin-dependent xenophagy of *Salmonella* inside a damaged SCV.
TI12 (25)	Inv, SCVdamage, T35, T33, T24, T22, T18, T20, T36, Syn5, Syn6, T25, T34, Deg3ii, T32ii, T21, T23, T19, Syn4, Syn3, T17, Syn2, T37, Syn9, Output	Ubquitin-dependent xenophagy of *Salmonella* inside the cytosol.
TI13 (27)	Inv, SCVdamage, T35, Deg3i, T33, T22, T20, T18, T36, Syn7, Syn5, Syn6, T34, T32i, T21, T23, T19, Syn4, Syn3, T26, T17, T37, Syn2, T27, Syn9, T28, Output	Ubquitin-dependent xenophagy of *Salmonella* inside the cytosol, including Nap1/Sintbad binding to NDP52.
TI14 (27)	Inv, SCVdamage, T35, Deg3i, T33, T24, T22, T20, T18, T36, Syn7, Syn5, T26i, Syn6, T25, T34, T32i, T21, T23, T19, Syn4, Syn3, T17, Syn2, T37, Syn9, Output	Ubquitin-dependent xenophagy of *Salmonella* inside the cytosol, including Nap1/Sintbad binding to NDP52.
TI15 (36)	Inv, SCVdamage, T35, Deg3, T33, T22, T20, T18, T36, Syn7, T29, Syn5, Syn6, T32, T34, T30', T31, T29', T30, T21, T23, T19, Syn4, Syn3, T17, T26, Syn2, T37, T27, 4*Syn8, Syn9, T28, Output	Ubquitin-dependent xenophagy of *Salmonella* inside the cytosol, including phosphorylation of OPTN.
TI16 (36)	Inv, SCVdamage, T35, Deg3, T33, T24, T22, T20, T18, T36, Syn7, T29, Syn5, T26i, Syn6, T25, T32, T34, T30', T31, T29', T30, T21, T23, T19, Syn4, Syn3, T17, Syn2, T37, 4*Syn8, Syn9, Output	Ubquitin-dependent xenophagy of *Salmonella* inside the cytosol, including phosphorylation of OPTN.
