# Supplementary material for: In Silico Knockout Studies of Xenophagic Capturing of Salmonella
Source: PLoS Comput Biol. 2016 Dec 1;12(12):e1005200. doi: 10.1371/journal.pcbi.1005200 (PMC5131900; doi:10.1371/journal.pcbi.1005200)
Supplement: S4 Table — (PDF) [file pcbi.1005200.s010.pdf]

S4 Table: P-invariants of the Petri net.

P-invariant ID (Size)	Places (Species)	Biological Meaning
PI1 (27)	Ap:Ub, S:Ub:NDP52:OPTN:p62:N/S:OligoTBK1, S:Ub:NDP52:OPTN:p62:N/S:diTBK1i, S:Ub:NDP52:OPTN:p62:N/S:diTBK1, NDP52', S:Gal8:Ub:NDP52, Ap:Ub:OPTNp, Ap:Ub:N/S, S:Gal8:NDP52, S:Ub:NDP52:OPTN:p62:N/S, S:Gal8:NDP52:Ub:OPTN:p62:N/S, S:Ub:NDP52:OPTN:p62, S:Gal8:Ub:NDP52:OPTN:p62, Ap:Gal8:Ub:OPTNp, Ap:Gal8, S:Ub:NDP52, S:Gal8:Ub:NDP52:p62:OPTNp:N/S:OligoTBK1, S:Ub:NDP52:N/S, NDP52, N/S'i, S:Gal8:Ub:NDP52:OPTN:p62:N/S:diTBK1, NDP52'i, S:Gal8:Ub:NDP52:OPTN:p62:N/S:diTBK1i, Ap:Gal8:Ub:N/S, Ap:Gal8:Ub, S:Gal8:Ub:NDP52:N/S, N/S'	Conservation of NDP52
PI2 (21)	Ap:Ub, Ap:Gal8:Ub:OPTNp, S:Ub:OPTN, S:NDP52:Ub:OPTN:p62:N/S:OligoTBK1, S:NDP52:Ub:OPTN:p62:N/S:diTBK1i, S:NDP52:Ub:OPTN:p62:N/S:diTBK1, OPTN'i, S:Gal8:NDP52:Ub:p62:OPTNp:N/S:OligoTBK1, Ap:Ub:OPTNp, OPTN', Ap:Ub:N/S, S:Gal8:NDP52:Ub:OPTN:p62:N/S:diTBK1, OPTN, S:Gal8:NDP52:Ub:OPTN:p62:N/S, S:NDP52:Ub:OPTN:p62:N/S, S:Gal8:Ub:OPTN S:NDP52:Ub:OPTN:p62, S:Gal8:NDP52:Ub:OPTN:p62, S:Gal8:NDP52:Ub:OPTN:p62:N/S:diTBK1i, Ap:Gal8:Ub:N/S, Ap:Gal8:Ub	Conservation of OPTN
PI3 (3)	AA, mTORC1inactive, AAstarvation	Conservation of the AA starvation signal
PI4 (4)	SignalAutophagyInduction, mTORC1:ULK1comp:SCV, ULK1comp, mTORC1:ULK1comp	Conservation of the ULK1 complex
PI5 (17)	Ap:Gal8:Ub:OPTNp, Ap:Gal8, Gal8, S:Gal8:Ub:NDP52, S:Gal8, S:Gal8:Ub:p62, S:Gal8:NDP52:Ub:p62:OPTNp:N/S:OligoTBK1, S:Gal8:NDP52, S:Gal8:NDP52:Ub:OPTN:p62:N/S:diTBK1, S:Gal8:Ub, S:Gal8:NDP52:Ub:OPTN:p62:N/S, S:Gal8:Ub:OPTN, S:Gal8:NDP52:Ub:OPTN:p62, S:Gal8:NDP52:Ub:OPTN:p62:N/S:diTBK1i, Ap:Gal8:Ub:N/S, Ap:Gal8:Ub, S:Gal8:Ub:NDP52:N/S	Conservation of galectin-8

PI6 (27)	Ap:Ub, S:NDP52:Ub:OPTN:p62:N/S:OligoTBK1, S:NDP52:Ub:OPTN:p62:N/S:diTBK1i, S:NDP52:Ub:OPTN:p62:N/S:diTBK1, S:Gal8:Ub:NDP52, LRSAM1, S:Gal8:Ub:p62, Ap:Ub:OPTNp, Ap:Ub:N/S, S:Gal8:Ub, S:NDP52:Ub:OPTN:p62:N/S, S:Gal8:NDP52:Ub:OPTN:p62:N/S, S:Gal8:Ub:OPTN, S:NDP52:Ub:OPTN:p62, S:Gal8:NDP52:Ub:OPTN:p62, S:Ub:OPTN, Ap:Gal8:Ub:OPTNp, S:Ub:p62, S:Ub:NDP52, S:Gal8:NDP52:Ub:p62:OPTNp:N/S:OligoTBK1, S:Ub:NDP52:N/S, S:Gal8:NDP52:Ub:OPTN:p62:N/S:diTBK1, S:Gal8:NDP52:Ub:OPTN:p62:N/S:diTBK1i, Ap:Gal8:Ub:N/S, Ap:Gal8:Ub, S:Gal8:Ub:NDP52:N/S, S:Ub	Conservation of LRSAM1
PI7 (27)	Ap:Ub, S:NDP52:Ub:OPTN:p62:N/S:OligoTBK1, S:NDP52:Ub:OPTN:p62:N/S:diTBK1i, S:NDP52:Ub:OPTN:p62:N/S:diTBK1, E3 ligase, S:Gal8:Ub:NDP52, S:Gal8:Ub:p62, Ap:Ub:OPTNp, Ap:Ub:N/S, S:Gal8:Ub, S:NDP52:Ub:OPTN:p62:N/S, S:Gal8:NDP52:Ub:OPTN:p62:N/S, S:Gal8:Ub:OPTN, S:NDP52:Ub:OPTN:p62, S:Gal8:NDP52:Ub:OPTN:p62, S:Ub:OPTN, Ap:Gal8:Ub:OPTNp, S:Ub:p62, S:Ub:NDP52, S:Gal8:NDP52:Ub:p62:OPTNp:N/S:OligoTBK1, S:Ub:NDP52:N/S, S:Gal8:NDP52:Ub:OPTN:p62:N/S:diTBK1, S:Gal8:NDP52:Ub:OPTN:p62:N/S:diTBK1i, Ap:Gal8:Ub:N/S, Ap:Gal8:Ub, S:Gal8:Ub:NDP52:N/S, S:Ub	Conservation of ubiquitin
PI8 (4)	SignalAutophagyInduction, AA, ULK1comp, AAstarvation	Conservation of the AA starvation signal
PI9 (21)	Ap:Ub, Ap:Gal8:Ub:OPTNp, S:NDP52:Ub:OPTN:p62:N/S:OligoTBK1, S:Ub:p62, S:NDP52:Ub:OPTN:p62:N/S:diTBK1i, S:NDP52:Ub:OPTN:p62:N/S:diTBK1, S:Gal8:Ub:p62, p62', S:Gal8:NDP52:Ub:p62:OPTNp:N/S:OligoTBK1, Ap:Ub:OPTNp, Ap:Ub:N/S, S:Gal8:NDP52:Ub:OPTN:p62:N/S:diTBK1, S:Gal8:NDP52:Ub:OPTN:p62:N/S, S:NDP52:Ub:OPTN:p62:N/S, p62'i, S:NDP52:Ub:OPTN:p62, S:Gal8:NDP52:Ub:OPTN:p62, S:Gal8:NDP52:Ub:OPTN:p62:N/S:diTBK1i, Ap:Gal8:Ub:N/S, Ap:Gal8:Ub, p62 Conservation of p62	
PI10 (6)	SCV, SignalAutophagyInduction, mTORC1:ULK1comp:SCV, SignalSCVdamage, ULK1comp, AAstarvation	Conservation of the SCV damage signal

PI11 (17)	Ap:Gal8:Ub:OPTNp, S:NDP52:Ub:OPTN:p62:N/S:OligoTBK1, S:NDP52:Ub:OPTN:p62:N/S:diTBK1i, S:NDP52:Ub:OPTN:p62:N/S:diTBK1, S:Gal8:NDP52:Ub:p62:OPTNp:N/S-OligoTBK1, Ap:Ub:OPTNp, Ap:Ub:N/S, S:Ub:NDP52:N/S, S:Gal8:NDP52:Ub:OPTN:p62:N/S:diTBK1, N/S'i, S:Gal8:NDP52:Ub:OPTN:p62:N/S, S:NDP52:Ub:OPTN:p62:N/S, N/S, S:Gal8:NDP52:Ub:OPTN:p62:N/S:diTBK1i, Ap:Gal8:Ub:N/S, S:Gal8:Ub:NDP52:N/S, N/S'	Conservation of Nap1/Sintbad
PI12 (3)	mTORC1:ULK1comp:SCV, mTORC1:ULK1comp, mTORC1inactive	Conservation of mTORC1
PI13 (32)	SCV, Ap:Ub, S:NDP52:Ub:OPTN:p62:N/S:OligoTBK1, S-cyt, S-damagedSCV, S:NDP52:Ub:OPTN:p62:N/S:diTBK1i, S:NDP52:Ub:OPTN:p62:N/S:diTBK1, S:Gal8, S:Gal8:Ub:NDP52, S:Gal8:Ub:p62, Ap:Ub:OPTNp, Ap:Ub:N/S, S:Gal8:NDP52, S:Gal8:Ub, S:Gal8:NDP52:Ub:OPTN:p62:N/S, S:NDP52:Ub:OPTN:p62:N/S, S:Gal8:Ub:OPTN, S:NDP52:Ub:OPTN:p62, S:Gal8:NDP52:Ub:OPTN:p62, S:Ub:OPTN, Ap:Gal8:Ub:OPTNp, S:Ub:p62, S:Ub:NDP52, Ap:Gal8, S:Gal8:NDP52:Ub:p62:OPTNp:N/S:OligoTBK1, S:Ub:NDP52:N/S, S:Gal8:NDP52:Ub:OPTN:p62:N/S:diTBK1, S:Gal8:NDP52:Ub:OPTN:p62:N/S:diTBK1i, Ap:Gal8:Ub:N/S, Ap:Gal8:Ub, S:Gal8:Ub:NDP52:N/S, S:Ub	Conservation of *Salmonella*
PI14 (35)	4*Ap:Gal8:Ub:OPTNp, 4*S:NDP52:Ub:OPTN:p62:N/S:OligoTBK1, 2*diTBK1'iii, 2*diTBK1'ii, 2*S:NDP52:Ub:OPTN:p62:N/S:diTBK1i, 2*S:NDP52:Ub:OPTN:p62:N/S:diTBK1, TBK1 4*S:Gal8:NDP52:Ub:p62:OPTNp:N/S:OligoTBK1, 4*Ap:Ub:OPTNp, 2*S:Gal8:NDP52:Ub:OPTN:p62:N/S:diTBK1, 2*diTBK1', 2*diTBK1'i, 2*S:Gal8:NDP52:Ub:OPTN:p62:N/S:diTBK1i	Conservation of TBK1
PI15 (5)	SCV, mTORC1:ULK1comp:SCV, SignalSCVdamage, mTORC1inactive, AAstarvation	Conservation of the SCV damage signal
