# Supplementary material for: In Silico Knockout Studies of Xenophagic Capturing of Salmonella
Source: PLoS Comput Biol. 2016 Dec 1;12(12):e1005200. doi: 10.1371/journal.pcbi.1005200 (PMC5131900; doi:10.1371/journal.pcbi.1005200)
Supplement: S5 Table — (PDF) [file pcbi.1005200.s011.pdf]

S5 Table: Single knockout and its impact on *Salmonella* xenophagy.

Single knockout	Number of affected T-invariants	Percentage of affected T-invariants
NDP52	16	100%
Ubiquitin/LRSAM1	15	94%
p62	15	94%
OPTN	15	94%
Nap1/Sintbad	12	75%
Galectin-8	11	69%
TBK1	6	38%
