# Supplementary material for: In Silico Knockout Studies of Xenophagic Capturing of Salmonella
Source: PLoS Comput Biol. 2016 Dec 1;12(12):e1005200. doi: 10.1371/journal.pcbi.1005200 (PMC5131900; doi:10.1371/journal.pcbi.1005200)
Supplement: S6 Table — (PDF) [file pcbi.1005200.s012.pdf]

S6 Table: Double knockout and its impact on *Salmonella* xenophagy.

**Double knockout**	**Number of affected T-invariants**	**Percentage of affected T-invariants**
Galectin-8, Ubiquitin/LRSAM1	16	100%
Galectin-8, p62	16	100%
Galectin-8, NDP52	16	100%
Galectin-8, OPTN	16	100%
Ubiquitin/LRSAM1, NDP52	16	100%
p62, NDP52	16	100%
NDP52, OPTN	16	100%
NDP52, Nap1/Sintbad	16	100%
NDP52, TBK1	16	100%
Ubiquitin/LRSAM1, p62	15	94%
Ubiquitin/LRSAM1, OPTN	15	94%
Ubiquitin/LRSAM1, Nap1/Sintbad	15	94%
Ubiquitin/LRSAM1, TBK1	15	94%
p62, OPTN	15	94%
p62, Nap1/Sintbad	15	94%
p62, TBK1	15	94%
OPTN, Nap1/Sintbad	15	94%
OPTN, TBK1	15	94%
Galectin-8, Nap1/Sintbad	15	94%
Galectin-8, TBK1	13	81%
Nap1/Sintbad, TBK1	12	75%
